# Primary sternal osteomyelitis: A case report

**DOI:** 10.1016/j.ijscr.2023.108654

**Published:** 2023-08-13

**Authors:** Amer Al Ani, Khadiga Abdelmonem, Kowthar Forsat, Nour Alqaderi, Hajar Teir

**Affiliations:** College of Medicine, Ajman university, Ajman, United Arab Emirates

**Keywords:** Sternum, Osteomyelitis, Debridement, *Staphylococcus aureus*, Case report

## Abstract

**Introduction:**

Primary sternal osteomyelitis is a rare condition that is frequently caused by *Staphylococcus aureus*. It is often confused with other cardiac and pulmonary conditions. Early antimicrobial treatment and surgical debridement is the cornerstone of treatment.

**Case presentation:**

A 51-year-old male adult came to the emergency room (ER) with a 2-week history of chest pain, fever, and malaise. His past medical history was unremarkable. Examination revealed a tender anterior chest wall swelling. White Blood Cells (WBCs) (21.6 × 10^4^)/mm^3^) and C-reactive protein (CRP) (294.10 mg/L) were elevated. Pus from the swelling and blood samples were sent for culture and sensitivity. Electrocardiogram (ECG) was normal and a computed tomography (CT) scan of the chest showed a large dense anterior chest wall abscess extending deep in the chest and to both axillae which caused bony erosion of the sternum. Incision and drainage of the abscess were performed, followed by surgical debridement of the wound. Cultures along the course showed both *Staphylococcus aureus* and Enterococcus. The patient improved gradually and 2 months after his initial presentation, he became free of symptoms, and CT has shown complete resolution.

**Discussion:**

Osteomyelitis usually happens after an external bacterium seeds the bone where it begins to grow and thrive, leading to the destruction and pus accumulation under the periosteum. For the treatment, identifying the causative agent is critical in giving intravenous (IV) antibiotic. Thereafter, incision and drainage of an abscess can be performed, similar to what was done with the patient mentioned. Radiography, specifically a CT scan, is crucial as it clearly reveals bony margins and can differentiate between a sequestrum and an involucrum. It also identifies cortical erosion, intraosseous gases and periosteal reactions.

**Conclusion:**

Sternal osteomyelitis can have a nonspecific clinical presentation. Laboratory investigations and radiological findings are crucial for a prompt diagnosis. To prevent the progression of the disease and complications, early intervention is vital to ensure a good prognosis.

## Highlights

• Primary sternal osteomyelitis is a rare condition.
• It is frequently caused by *Staphylococcus aureus*.
• It is often confused with other cardiac and pulmonary conditions.
• Early antimicrobial treatment and surgical debridement is the cornerstone of treatment.

## 1 Introduction

This work has been reported in line with the SCARE criteria [1].

Sternal osteomyelitis usually presents with chest pain, fever, tenderness, and swelling. However, some of these signs and symptoms might not be present and sternal osteomyelitis may be confused with other cardiac and pulmonary conditions [2]. Since sternal osteomyelitis is not common, it can be initially misdiagnosed, thus delaying its management [2]. Radiological imaging, microbiology testing and other laboratory modules help diagnose suspected cases of osteomyelitis [3]. CT and magnetic resonance imaging (MRI) are currently considered the standard for diagnosing osteomyelitis [4]. Radiological evidence of sternal osteomyelitis may take weeks to appear after the start of infection; however, physicians should not wait until radiological evidence is present if clinical signs and other lab results confirm sternal osteomyelitis. The most common bacterial cause of sternal osteomyelitis is *Staphylococcus aureus*, meanwhile *Pseudomonas aeruginosa* is common in IV drug users [5]. Treatment of sternal osteomyelitis can be initiated after identifying the causative microorganism, which is determined by blood culture or direct bone biopsy [5]. Antibiotics are the primary treatment of sternal osteomyelitis, but with the addition of surgical debridement, antibiotic treatment has been proven to be more efficacious [6]. Surgical intervention is necessary in cases where antibiotic treatment alone has failed or when abscesses and extensive bone necrosis are present [6].

## 2 Case presentation

A 51-year-old male prisoner presented to ER with chest pain that started two weeks ago and has worsened progressively. At the presentation, he was ill-looking and distressed but was alert, well-oriented, and cooperative. On physical examination, a bilateral, tender swelling on the anterior chest wall was present that started two weeks prior. This swelling was fluctuating and erythematous with no discharge. He also had supple, nontender cervical lymph nodes, otherwise the rest of the physical examination was unremarkable.

Additionally, there was cellulitis on the medial aspect of the distal tibia and ankle. His skin looked warm, pink and moist. Upon admission, his vital signs were within normal limits. The patient was admitted to the hospital and aspiration from chest swelling was done. On needle aspiration, pus was present. Pus culture showed significant growth of *Staphylococcus aureus* which was sensitive to Linezolid and amoxicillin/clavulanate. Blood culture showed the same organism. Complete blood count (CBC) revealed WBC count of 21.6 × 10^3/^mm^3^, neutrophils of 86.10 %, hemoglobin (Hb) was 12.90 g/dL, platelets were 389.00 × 10^9^/mm^3^. C-reactive protein (CRP) was 294.10 mg/L. Alanine transaminase (ALT): 236 IU/L, alkaline phosphatase (ALP): 185 IU/L, total protein of 81 g/dL, albumin of 19.2 g/dL, bilirubin of 28.9, lactic acid of 4.8 (high). Prothrombin time (PT) was prolonged to 16.20 s. (ECG) was within normal. On admission a parenteral antibiotic was started empirically. Chest (CT) scan illustrated a large multiseptated dense anterior chest wall lesion, extending deep to both pectoralis major muscles, neck root, axillae, and anterior mediastinal leading to bony erosion of the sternum.

An incision was made over the chest wall swelling. Large amount of seropurulent fluid was drained, debridement of all necrotic areas in the subcutaneous layer, fascia, medial portion of pectoralis major muscles, and sternum was done. A vacuum dressing was applied and the patient was continued on the parenteral antibiotic.

The vacuum dressing was changed every few days. A follow-up CT showed bilateral pectoralis major fluid collection measuring 8 × 5.6 × 4 cm that was connected to the midline chest wound, indicating an abscess on the apical-lateral chest wall, and there was a retrosternal enhancing soft tissue mass, without any fluid collection. The CT also showed destructive sternal changes, bilateral small pleural effusion, and bilateral atelectatic changes, reactionary to sternal osteomyelitis.

**Fig. 1:**
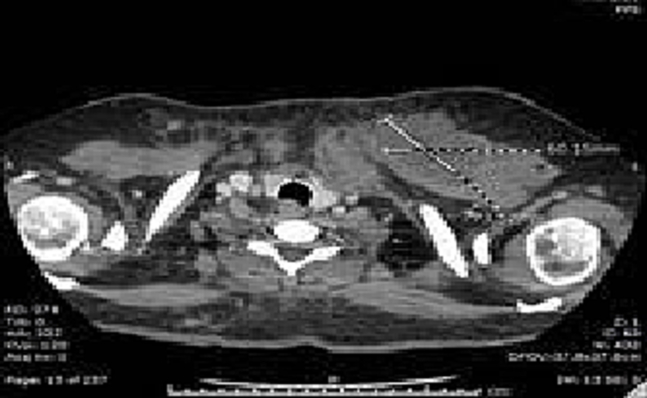
Left infraclavicular abscess following sternal osteomyelitis is shown.

**Fig. 2:**
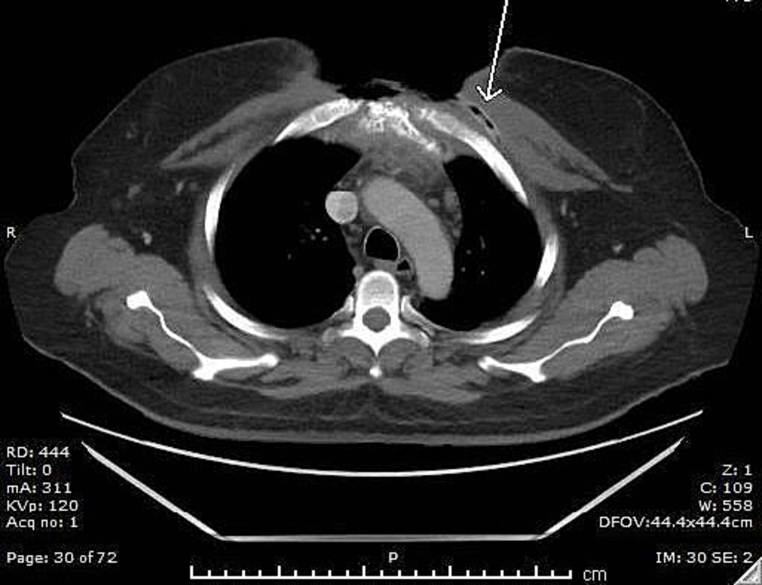
Sternal bone shows destruction, cortical erosion, and inhomogeneous density. Retrosternal space is showing fluid collection, fat stranding, and inflammatory changes. Subcutaneous air loculi along the incisional line (arrow).

Following debridement and vacuum dressing application, a healthy granulation tissue started to cover the wound. A follow-up CT scan (Fig. 1) showed residual collection (measuring 70 × 60 × 34 mm) confined to the superior aspect of the left pectoralis major. There was still a retrosternal enhancing soft tissue swelling without fluid collection. Another debridement was done. Wound culture showed Enterococcus and *Staphylococcus aureus*, which were sensitive to Linezolid and amoxicillin/clavulanate. A follow-up CT scan, and in comparison, (Fig. 2) to the previous CT scan, revealed collapse and resolution of the previous left chest wall abscess, with clear lung fields and no other significant internal changes.

Six weeks later, after frequent debridement and vacuum dressing application, the vacuum was removed and the patient was discharged with instructions to have daily dressing with the plan to have skin graft after maintaining a clean wound. The patient was lost for follow-up.

## 3 Discussion

Osteomyelitis usually happens after external bacterium seeds sternum bone where it begins to grow and thrive, leading to destruction and pus accumulation under periosteum [2,3]. Depending on the etiology, sternal osteomyelitis is classified into primary, presented in this case, and secondary types [6]. Risk factors include obesity, HIV, and diabetes mellitus among others [5,7]. However, there are documented cases with none of these risk factors [6,8]. Identifying the causative agent is critical in giving IV antibiotic [2]. In 2022 a review by Cha, Y. K., Et al. found that Staphylococcus was the most common organism in sternal wound cultures [8]. Our patient's sternum was infected with *Staphylococcus aureus* despite treatment with antibiotics. The infection kept recurring resulting in repeated wound debridement procedures and the patient losing parts of his sternum bone. As the infection extended to the mediastinum, resulting in a mediastinal abscess whose culture was positive for *Enterococcus faecalis*. The seeding of the bacteria occurred because of direct inoculation from the skin infection. Our patient was a prisoner, which means it could have likely been Methicillin-resistant *Staphylococcus aureus* [MRSA] [9]. According to a review by Yusuf E. Et al., the reported incidence of deep sternal wound infection is low; however, it has adverse effects that lead to increased costs and longer hospital stays [10].

The diagnosis of primary sternal osteomyelitis is based on clinical presentation, radiologic imaging, laboratory, and microbiological findings. However, the diagnosis is usually delayed due to few reported cases and nonspecific clinical presentation. Sternal bony pain, painful chest mass, tenderness, swelling, and relapsing fever for more than 10 days, with any of the risk factors, should raise the suspensions of PSO. However, these symptoms might not be present in all cases and may be confused with other conditions [5]. Other differentials include 1) Cardiac causes (like: Heart valve disease, hypertrophic cardiomyopathy, coronary artery disease, myocarditis, pericarditis and aortic dissection 2) Skin and soft tissue infections (SSTIs) or tumors, and 3) Musculoskeletal diseases [11]. Our patient was symptomatic; initially presented with progressive chest pain, tenderness, swelling, and redness.

Images used in the diagnosis of PSO, include MRI and bone scan. Both have high sensitivity and specificity, but the first imaging modality is conventional radiography. It shows soft tissue swelling as subtle sclerosis around the manubriosternal junction. However, bone destruction starts and can only be seen around 10–21 days after infection, before that period of time, the x-ray won't show a positive finding [12].. Ultrasound is mainly used to evaluate soft tissue, or as a guide for diagnostic, therapeutic aspiration, and tissue biopsy [13]. The laboratory findings are usually nonspecific; however, they can be helpful. For example, CRP, ESR and WBCs are among the first to rise; they were initially increased in our patient and remained elevated throughout [14]. Ca, Po4 and ALP, which was around 185 IU/L, can be used to exclude metastatic and metabolic bone diseases since they are usually normal in osteomyelitis [11]. Blood culture of sternal osteomyelitis can be negative, nonetheless, in our case, it was positive for *Staphylococcus aureus* [11]. Bone biopsy provides a definitive diagnosis as in this case and is recommended whenever feasible [15].

There is no consensus on the standards of care for sternal osteomyelitis because of the limited literature discussing this topic. Management differs depending on the clinical presentation, whether the sternal infection is superficial or deep, and culture results. However, the basic principles of treatment usually include antibiotics, drainage of pus, surgical debridement of necrotic tissue, and different closure techniques [6,10]. Antibiotics, specifically that cover *Staphylococcus aureus*, are usually enough to treat sternal osteomyelitis [6,16]. However, surgical intervention is necessary in cases where antibiotic treatment alone has failed or when abscesses and extensive bone necrosis are present [17]. Early surgical intervention usually ensures definitive treatment, decreases morbidity, and is more cost-effective. The surgical management usually include complete debridement of the infected bone and the anterior periosteum. The anterior bone defect is generally covered with a vascularized muscle flap [17] while the posterior periosteum is kept facilitating osteogenesis and sternal healing. Vacuum-associated dressing is applied to remove exudate, accelerate wound healing and is associated with better outcomes [10]. Treatment failure can cause mediastinitis, abscess collection, chronic infection, fistulae, and sinus tracts formation [2]. In our case, the patient was treated with surgical drainage of chest wall abscess, was put on cloxacillin-clindamycin, underwent multiple debridement procedures, and was put on vacuum dressing.

We emphasize the importance of early detection and optimum treatment of patients with sternal osteomyelitis to avoid complications. Broad spectrum empiric antibiotic regimen should be used, covering both gram-negative and gram-positive organisms [18], in order to avoid reinfection of the wound with other organisms.

## 4 Limitations

Since this is a case report, future studies must include larger samples in order to reach stronger conclusions. In like manner, there is limited literature and no unified guidelines discussing the management of primary sternal osteomyelitis.

Informed consent: Written informed consent was obtained from the patient for publication and any accompanying images. A copy of the written consent is available for review by the Editor-in-Chief of this journal on request.

## Institutional review board or ethical committee approval

Ethics clearance was not necessary, as this study is a case study.

## Research registration

Research registry 9245, Registration Date: July 08, 2023 22:49 and the hyperlink to registration: http://www.researchregistry.com

## Declaration of competing interest

Nothing to declare. This study did not receive any funding.
